# A brainstem-central amygdala circuit underlies defensive responses to learned threats

**DOI:** 10.1038/s41380-019-0599-6

**Published:** 2019-11-22

**Authors:** Yiran Gu, Walter T. Piper, Lauren A. Branigan, Elena M. Vazey, Gary Aston-Jones, Longnian Lin, Joseph E. LeDoux, Robert M. Sears

**Affiliations:** 1 Shanghai Key Laboratory of Brain Functional Genomics (Ministry of Education), Institute of Brain Functional Genomics, School of Life Science, NYU-ECNU Institute of Brain and Cognitive Science, East China Normal University, Shanghai, China; 2 Center for Neural Science, New York University, New York, NY USA; 3 Department of Psychiatry, McLean Hospital, Harvard Medical School, Belmont, MA USA; 4 Department of Biology, University of Massachusetts, Amherst, MA USA; 5 Brain Health Institute, Rutgers University/Rutgers Biomedical and Health Sciences, Piscataway, NJ USA; 6 Emotional Brain Institute, The Nathan Kline Institute, Orangeburg, NY USA; 7 Department of Child and Adolescent Psychiatry, New York University Langone School of Medicine, New York, NY USA

**Keywords:** Psychology, Neuroscience

## Abstract

Norepinephrine (NE) plays a central role in the acquisition of aversive learning via actions in the lateral nucleus of the amygdala (LA) [1, 2]. However, the function of NE in expression of aversively-conditioned responses has not been established. Given the role of the central nucleus of the amygdala (CeA) in the expression of such behaviors [3–5], and the presence of NE axons projections in this brain nucleus [6], we assessed the effects of NE activity in the CeA on behavioral expression using receptor-specific pharmacology and cell- and projection-specific chemogenetic manipulations. We found that inhibition and activation of locus coeruleus (LC) neurons decreases and increases freezing to aversively conditioned cues, respectively. We then show that locally inhibiting or activating LC terminals in CeA is sufficient to achieve this bidirectional modulation of defensive reactions. These findings support the hypothesis that LC projections to CeA are critical for the expression of defensive responses elicited by conditioned threats.

## Introduction

Much of the work describing the neural and behavioral mechanisms of defensive behavior and threat processing has used Pavlovian threat conditioning (PTC) [7]. This research has shown that the amygdala plays a crucial role in defensive reactions initiated by environmental threats [8, 9]. PTC and the amygdala have both been implicated in fear and anxiety disorders [8, 10], as has the neuromodulator norepinephrine (NE). In the present study, we explore the contribution of NE in the amygdala to the expression of amygdala controlled defensive behavior. During PTC, a neutral conditioned stimulus (CS; e.g., an acoustic tone) is paired with a noxious unconditioned stimulus (US; e.g., an electric foot shock) so that later presentation of the CS alone results in expression of defensive behaviors (the conditioned response; e.g., freezing). CS and US signals converge in both the lateral nucleus of the amygdala (LA) and the central nucleus of the amygdala (CeA) [8, 11–13]. The LA communicates directly with CeA [14] and indirectly via the basal amygdala (BA) [12, 14, 15]. As a major output nucleus of the amygdala, the CeA coordinates defensive behavioral reactions and supports physiological adjustments in response to threatening stimuli via divergent projections to the midbrain [4, 16–19], lateral/paraventricular hypothalamus (PVN) [18, 20, 21], and medulla [3, 16, 22].

NE is also implicated in fear and anxiety [23, 24]. Aversive stimuli and stress increase levels of NE in the brain, including the amygdala [25], largely through activation of the brain stem locus coeruleus (LC) [26, 27]. LC stimulation or noxious stimuli (e.g., footshock) modulate the basolateral region of the amygdala (BLA), which consists of LA and BA [25, 26, 28], and studies suggest that this is through direct NE activity at β-adrenergic receptors (β-ARs) [2, 29–32]. Notably, β-ARs in the LA are critical for initial acquisition (and indirectly for consolidation [2] processes), but not expression of Pavlovian threat memories [1, 2], and in BLA, for conditioned place aversion and anxiety-like behaviors [33]. Although much work on the role of NE in the amygdala has focused on the BLA, the CeA also receives significant NE inputs from the LC [34–37]. Despite this anatomical evidence, few studies have examined the contribution of NE inputs to CeA to the expression of defensive responses elicited by conditioned threats.

Here we describe the role of NE in the expression of Pavlovian threat memories and uncover key components of the underlying brain circuitry. We first show that CS-elicited defensive responses (freezing) decreased following systemic injection of the β-AR antagonist (propranolol), while injection of β_2_-AR agonist (procaterol) increased freezing. To test the role of LC in these adrenergic effects on behavioral expression, we used adeno-associated virus (AAV) vectors expressing DREADDs [38] and engineered to target NE-expressing neurons in the LC (NE-LC) [21, 39]. Systemic Clozapine-N-oxide (CNO) injections administered prior to testing reduced or enhanced behavioral expression, by inhibiting (hM4Di) or activating (hM3Dq) NE-LC neurons, respectively. To test the hypothesis that CeA mediates these effects, we directly infused propranolol into the CeA, which also reduced freezing. To test the role of a specific NE-LC→CeA circuit in expression, we directly inhibited or activated LC axon terminals by infusing CNO into the CeA prior to the expression test. Consistent with the pharmacology and NE-LC DREADD studies, we found that inhibition and activation of LC terminals in CeA bidirectionally modulated freezing behavior. Finally, to test a requirement for β-AR activation in the circuit-specific hM3Dq results, propranolol was co-infused with CNO in CeA. As predicted, propranolol attenuated CNO-induced enhancement of freezing. Taken together, these studies demonstrate that NE released from LC terminals in CeA enhances the expression of defensive responses elicited by learned threats.

## Methods

### Subjects

We used adult male Sprague-Dawley rats (Hilltop Laboratory Animals, Inc.; Scottdale, PA, USA), weighing 250–275 g (~60–70 days old) upon arrival. All animals were naive and allowed at least 1 week of acclimation to the vivarium before surgery and conditioning. Rats were individually housed in transparent plastic high-efficiency particulate absorption (HEPA)-filtered cages and maintained on a 12/12 h light/dark cycle (7:00 A.M.–7:00 P.M.) within a temperature- and humidity-controlled environment. Food and water were available *ad libitum* throughout the duration of the experiments. All experiments were conducted during the light cycle. All procedures were conducted in accordance with the *National Institutes of Health Guide for the Care and Use of Experimental Animals* and were approved by the *New York University Animal Care and Use Committee*.

### Stereotaxic surgery

Rats were anesthetized with a mixture of ketamine (100 mg/kg, i.p.) and xylazine (10 mg/kg, i.p.), and placed in a stereotaxic apparatus (David Kopf Instruments, Tujunga, CA, USA). Supplemental doses of the mixture were given as needed to maintain a deep level of anesthesia. Brain areas were targeted using coordinates from Paxinos and Watson [40]. Following surgery, rats were administered three daily doses of ketoprofen (5.0 mg/kg) as analgesic.

For CeA/BLA infusion experiments, rats were implanted bilaterally with stainless steel guide cannulae (22 gauge; Plastics One, Roanoke, VA, USA). Guide cannulae were lowered to CeA (stereotaxic coordinates from bregma: anterior–posterior (AP) –2.8 mm, medial–lateral (ML) ± 4.3 mm, dorsal–ventral (DV) –7.0 mm from skull) or BLA (stereotaxic coordinates from bregma: anterior–posterior (AP) –2.8 mm, medial–lateral (ML) ± 5.2 mm, dorsal–ventral (DV) –7.0 mm from skull) and secured to the skull using surgical screws and acrylic dental cement (Ortho-jet; Lang Dental Manufacturing Co.). Dummy cannulae (28 gauge) extending 0.2 mm from the guides were inserted to prevent clogging. Internal cannulae (28 gauge) extending 1.5 mm beyond the guides were used for drug infusions. Results of cannula targeting are shown in Supplementary Fig. 1.

For LC viral injection experiments (Supplementary Fig. 2), DREADD virus was bilaterally injected (stereotaxic coordinates from lambda: anterior–posterior (AP) –0.8 mm, medial–lateral (ML) ± 1.35 mm, dorsal–ventral (DV) –7.5 mm from skull) to a volume of 1.4 µl/side using a 5.0 µl Hamilton Neuros syringe (Hamilton Co.). After 3 weeks for LC soma manipulations or 6–8 weeks for LC axon manipulations in CeA or BLA, animals were handled and subjected to behavioral conditioning as described below.

### Apparatus

For behavioral experiments, rats underwent threat conditioning in one of six identical chambers (Rat Test Cage; Coulbourn Instruments, Allentown, PA, USA) constructed of aluminum and Plexiglas walls, with metal stainless steel rod flooring that was attached to a shock generator (Model H13-15; Coulbourn Instruments). Each chamber was enclosed within a sound isolation cubicle (Model H10-24A; Coulbourn Instruments). A computer, installed with Graphic State 2 software and connected to the chambers via the Habitest Linc System (Coulbourn Instruments), delivered tone and shock stimuli during behavioral sessions. During habituation and threat conditioning, the chambers were lit with a single house light (context A). Expression tests took place in a modified context which consisted of red lighting, smooth black plastic flooring, a mild peppermint, or lavender scent and a striped pattern on the Plexiglas door (context B or context C), a mild almond scent or a mild citrus scent and a striped pattern on the Plexiglas door (context D or context E). An infrared digital camera mounted on top of each chamber was used to videotape behavioral procedures.

### Viral vectors

Excitatory (hM3Dq: AAV9/PRS × 8-HA-hM3Dq-SV40-PolyA), inhibitory (hM4Di: AAV9/PRS × 8-HA-hM4Di-SV40-PolyA) and control vectors (Control: AAV9/PRS × 8-mCherry-WPRE-rBG) were subcloned by Dr. Elena M. Vazey from Gary Aston-Jones’ lab and packaged by the University of Pennsylvania Vector Core. The synthetic PRS × 8 promoter was used to restrict expression of the hM3Dq/hM4Di DREADDs to noradrenergic neurons in the LC (see Supplementary Fig. 2).

### Drug preparation and Infusion

(±)-Propranolol hydrochloride and procaterol hydrochloride (Sigma-Aldrich Co. St. Louis, MO, USA) were freshly dissolved in 0.9% sterile saline immediately prior to injections. For intraperitoneal (IP) injection experiments, concentrations for propranolol and procaterol were 10 mg/kg and 300 µg/kg, respectively. For CeA microinfusion experiments, propranolol was dissolved in 0.9% sterile saline and administered at 1.0 µg/0.3 µl. CNO for IP experiments (5.0 mg/kg for hM4Di inhibition, 1.0 mg/kg for hM3Dq activation) was obtained from the NIH as part of the Rapid Access to Investigative Drug Program funded by the NINDS and prepared in a 7% DMSO + 0.9% sterile saline, which was also used for vehicle. For c-Fos experiments, CNO was obtained from RTI International (Batch ID: 13662-18; MH No. C-929). For intracranial CeA or BLA infusions, CNO (Sigma-Aldrich) was dissolved in 0.9% sterile saline (1.0 mM, 0.3 μl per side) [41].

For all intracranial infusions, infusion cannulae were attached to 10 µl Hamilton syringes via 0.015 in. × 0.043 in. × 0.014 in. polyethylene tubing obtained from A-M Systems, Inc. (Carlsborg, WA, USA). Tubing and syringes were backfilled with distilled water, and a small air bubble was introduced to separate the water from the infusate. Rats were bilaterally infused with 0.3 µl using an infusion pump (PHD 2000; Harvard Apparatus) at a constant rate of 0.1 µl/min. Animals were allowed to move freely in their home cage during infusions. After infusion was complete, cannulae were left in place for an additional 1–2 min to allow drug diffusion away from the cannula tip.

For infusions to estimate spread of drugs in CeA, a fluorophore-conjugated propranolol (CA200693 CellAura fluorescent β_2_ antagonist [(S)-propranolol-green], Hello Bio Inc., Princeton, NJ, USA) was dissolved at a concentration of 0.5 μg/μl in a vehicle of 5% DMSO, 25% TWEEN 80, and 70% by volume of saline. Animals were infused as described above and were perfused with 4% paraformaldehyde 30 min after fluorophore infusion.

### Pavlovian threat conditioning and testing procedures

Rats were habituated to the conditioning box (context A) for 30 min and returned to the colony room. Twenty-four hours following habituation (Day 2) animals were threat conditioned in the same context. Following an initial 5-min acclimation period, rats were subject to three conditioning trials consisting of a 30-s, 5 kHz, 80 dB SPL sine-wave tone CS co-terminating with a 1-s footshock US (a 0.4 mA weak footshock US, a 0.6 mA standard footshock US, or a 1.0 mA strong footshock (see Supplementary Fig. 4)). Expression testing for CS-elicited freezing responses was conducted 1 day after conditioning in the modified context (context B). After the 5-min acclimation period, rats were presented with five CS presentations without the footshock US. The mean inter-trial interval was 4 min (2–6 min range) for both conditioning and testing sessions. On Day 4, 2 days after conditioning, drug-free testing for CS-elicited freezing was conducted in a modified context (context C) using the same procedure as the previous expression test. Behavior was recorded and freezing scored as described below.

### Measurement of freezing behavior

Freezing was used to measure the conditioned threat response and was defined as the cessation of all movement with the exception of respiration-related movement and non-awake or resting body posture [42]. Behavior was recorded and scored offline as the time spent freezing during each 30-s tone CS. Pre-CS freezing was also scored during the 30-s interval prior to the initial tone onset and was used as a measure of non-specific freezing to the context. Two experimenters, blind to drug group allocation scored freezing and the data were averaged.

### c-Fos experiments to confirm DREADD activity

Some animals used for DREADD experiments were injected IP with CNO or saline to validate the functionality of DREADDs (Supplementary Fig. 5a and 5b). Animals expressing excitatory hM3Dq receptors were injected IP with either CNO (1.0 mg/kg) or saline, then perfused 90 minutes later with 4% paraformaldehyde (PFA) as described in the histology section below. Animals expressing inhibitory hM4Di were injected IP with CNO (5.0 mg/kg) or saline, then 30 minutes later exposed to footshocks by running the CS-US conditioning procedure again (to increase baseline c-Fos levels) and perfused 85 minutes after completion of the three tone-footshock pairings. Brain sections and microscope images were prepared as described below for c-Fos counts of cell nuclei in LC.

### Histology and immunohistochemistry

Following behavior experiments, animals were overdosed with 25% chloral hydrate or a mixture of ketamine (100 mg/kg, i.p.) and xylazine (10 mg/kg, i.p.) and transcardially perfused with either 10% formalin for histology to assess cannula placement or 4% PFA in 0.1 M phosphate buffer (PB) for immunohistochemistry (IHC).

Tissue processed for cannula placement was post-fixed in 10% formalin at 4 °C until prepared for histological staining. For IHC, some brains were cryoprotected in a 30% sucrose–4% PFA solution for at least 1 day and then stored in 0.01 M PBS at 4 °C before being sectioned on a freezing microtome (Leica). Other brains were post-fixed in 4% PFA, blocked coronally, and cut on a Vibratome (Leica).

For histological verification of cannula targeting, tissue was cut at a thickness of 50 μm and kept in 0.01 M PBS + 0.05% sodium azide (NaAz) until mounted on gelatin-coated slides and dried overnight. After standard Nissl staining and coverslipping, sections were examined on a light microscope for injector tip localization in the CeA or BLA. Only data from rats with bilateral injector placements localized to the CeA or BLA were included in the study.

For IHC, tissue was cut at 35 or 40 μm, rinsed in 0.01 M PBS, and blocked in 1% BSA in 0.01 M PBS for 30–60 min at room temperature (RT). Immunohistochemical detection was achieved in primary antibody solutions containing 1% BSA, 0.2% Triton-X 100, and 0.05% NaAz.

DREADD-injected brain sections were incubated overnight at RT in rabbit anti-HA (for detection of HA-Tag; 1:500; cat. # 3724s; Cell Signaling Technology, MA) and mouse anti-dopamine beta hydroxylase (DBH; 1:2000; cat. # MAB308; EMD MIllipore, MA) antibodies for verification of viral expression in LC neurons, long-projection terminals in CeA, and cell specificity of viral expression. Viral control animal brain sections were incubated overnight at RT in rabbit anti-DsRed (for detection of mCherry; 1:500; cat # 632496; Clontech Laboratories, CA) and anti-DBH (DBH; 1:2000; cat. # MAB308; EMD Millipore, MA) antibodies. For c-Fos experiments, guinea pig anti-c-Fos (1:1000; cat. # 226-005; Synaptic Systems, Goettingen, Germany) was used to detect c-Fos in LC cell nuclei.

Following primary antibody incubation, sections were rinsed with agitation three times for 5 min in 0.01 M PBS at RT. Sections were then incubated in Alexa Fluor goat anti-rabbit 594 (1:200; cat. # A-11012; Life Technologies, CA) or goat anti-mouse 488 secondary antibody (1:200; cat. # A-11001; Life Technologies, CA) in 0.01 M PBS at RT. For c-Fos experiments, secondary antibodies were Alexa Fluor goat anti-rabbit 488 (1:400; Thermo Fisher Scientific, MA) to label HA, Alexa Fluor goat anti-mouse 555 (1:800; Thermo Fisher Scientific, MA) to label DBH, and Alexa Fluor goat anti-guinea pig 647 (1:400; cat. # A-21450; Thermo Fisher Scientific, MA, USA) to label c-Fos. Sections were rinsed three times for 5 min in PBS, mounted on gelatin-coated slides, and allowed to dry for several hours, followed by a brief wash in deionized H_2_O to remove excess salt (PBS), coverslipped in aqueous mount (ProLong Gold Antifade Reagent; cat. #3P6930 or #3P6931; Life Technologies, CA), and allowed to cure overnight at RT. Sections were imaged using a Leica TCS SP8 confocal microscope (Leica) or Olympus VS120 fluorescent microscope (Olympus). Imaging data were processed and analyzed with ImageJ software (NIH). For all experiments, animals were excluded from analysis if virus-expression was insufficient or cannulae targeting was outside the areas of interest.

c-Fos and HA-expressing cells in LC were quantified by two raters blind to experimental condition. Images were prepared by importing confocal *z*-stacks into ImageJ software, which were then partially collapsed to project 10 *z*-planes to produce an image representing a 6.8 μm thick plane of LC. The measure of interest was the ratio of HA + c-Fos double-labeled cells over HA cells in LC.

### Statistics

Rats were randomly allocated to groups prior to surgery and behavioral testing. Previous studies using similar techniques guided our estimates of sample size. Out of 382 total animals, 103 were removed for these reasons, leaving 279 animals for analysis. Animals were excluded from the study if (1) virus expression was absent unilaterally or bilaterally, (2) if cannulae missed their target (bilaterally or unilaterally) and (3) if baseline freezing was not significantly different from CS-elicited freezing, indicating contextual generalization. For experiments with two groups, an unpaired Student’s *t* test (two-tailed) was used to analyze freezing levels (baseline or CS-elicited freezing). One-way ANOVA was used for comparing more than two groups followed by a Tukey’s multiple comparison tests. Mean CS freezing data between drug-treatment days and drug-free days were analyzed using repeated measures ANOVAs followed by Sidak multiple comparisons tests. For all experiments, normal distribution was assumed. A Brown–Forsythe test for equal variances was used for one-way ANOVAs, whereas the *F* test was used for the Student's *t* test to confirm that variances were not significantly different in compared groups. Where unequal variances were revealed by *F* test, data was reanalyzed using a Mann–Whitney *U* test. All behavioral results were replicated in multiple groups or runs for each experiment. Error bars in all figures represent ± SEM. Data were analyzed using GraphPad Prism.

## Results

**Fig. 1:**
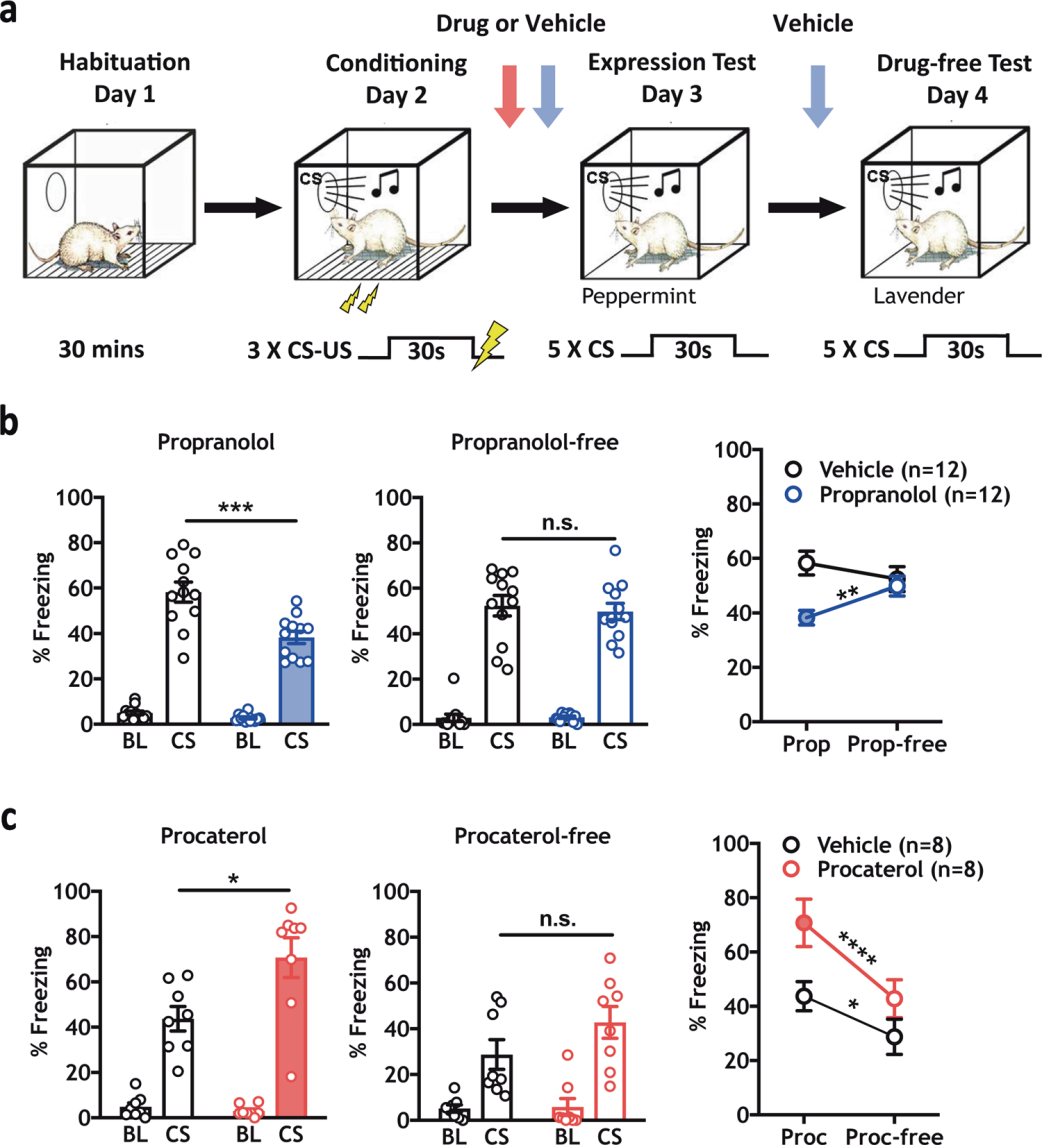
Norepinephrine β-AR activity is required for CS-elicited freezing responses. **a** Experimental timeline depicting habituation (Day 1), training (0.6 mA or 0.4 mA US) (Day 2), expression test (Day 3) and drug-free test (Day 4) phases. Vertical arrows indicate time of drug (red arrow) or vehicle (blue arrow) injection for each manipulation. **b** Systemic injection of the β-AR antagonist propranolol (10 mg/kg) reduced baseline (*p = 0.0283) and CS-elicited (***p = 0.0008) freezing levels during the expression test compared with vehicle control animals (left panel), with no effect observed between groups during a drug-free test (center panel). A within-subject comparison of propranolol treatment versus propranolol-free treatment on CS-elicited freezing showed a significant difference for drug treatment (two-way RM ANOVA test, Interaction: *F* (1, 22) = 15.79, ***p = 0.0006; Time (Drug versus Drug-free) *F* (1,22) = 1.678, *p* = 0.2086; Drug versus Vehicle *F* (1, 22) = 5.021, *p = 0.0355; Sidak MCS, **p < 0.01 between days for propranolol treated animals, n.s. for vehicle-treated animals). **c** Systemic injection of the specific β_2_-AR agonist procaterol (300 µg/kg) enhanced CS-elicited freezing during the expression test (*n* = 8/group; left panel, *p = 0.0200), with no effect during a drug free test (center panel). Within subject analysis showed a main effect of procaterol on CS-elicited freezing between days in both groups (two-way RM ANOVA test, Interaction: *F* (1, 14) = 3.991, *p* = 0.0655; Drug: *F* (1, 14) = 4.786, *p = 0.0462; Time (Drug versus Drug-free), *F* (1, 14) = 43.73, ****p < 0.0001; Sidak MCS, ****p < 0.0001 between days for procaterol treated animals, *p < 0.05 for vehicle-treated animals). All error bars indicate mean ± SEM. *p < 0.05, **p < 0.01, ***p < 0.001, ****p < 0.0001

We first investigated the contribution of β-AR activity to the expression of Pavlovian conditioned defensive responses. Rats were administered the β-AR antagonist propranolol (10 mg/kg, *n* = 12) or saline (*n* = 12) 24 h following cued threat conditioning and tested for freezing responses to the CS (Fig. 1a). Consistent with another report [43], propranolol significantly attenuated CS-evoked freezing levels (*t* (22) = 3.894, ***p = 0.0008), and slightly reduced baseline freezing (*t* (22) = 2.348, *p = 0.0283) (Fig. 1b, left panel), effects that were not observed during a drug-free test in the same animals (Fig. 1b, center panel). In separate groups of animals, propranolol still reduced freezing with stronger training (Supplementary Fig. 4a, 4b, *n* = 11/group, 1.0 mA shock training (*t* (20) = 2.129, *p = 0.0459)) and during a remote memory test (Supplementary Fig. 4c, 4d, *n* = 9/group, 1 month after conditioning (BL freezing: Student’s *t* test, *t* (16) = 2.297, *p = 0.0354, CS freezing: *t* (16) = 5.930, ****p < 0.0001).

To assess the effects of β_2_-AR activation on memory expression, two groups of rats were administered systemic injections of the β_2_-AR agonist procaterol (300 µg/kg, *n* = 8) or vehicle (*n* = 8) prior to the expression test. Procaterol significantly increased CS-evoked freezing levels (Fig. 1c left panel, 300 µg/kg, (t (14) = 2.625, *p = 0.0200)), with no difference observed between groups when the drug was not onboard (Fig. 1c, center panel) . Collectively, these data reveal that β-ARs positively modulate the expression of defensive responses regardless of memory strength or time since memory formation, and blockade or activation does not have long-term effects on behavioral plasticity.

### Chemogenetic inhibition of LC attenuates freezing to a conditioned cue

**Fig. 2:**
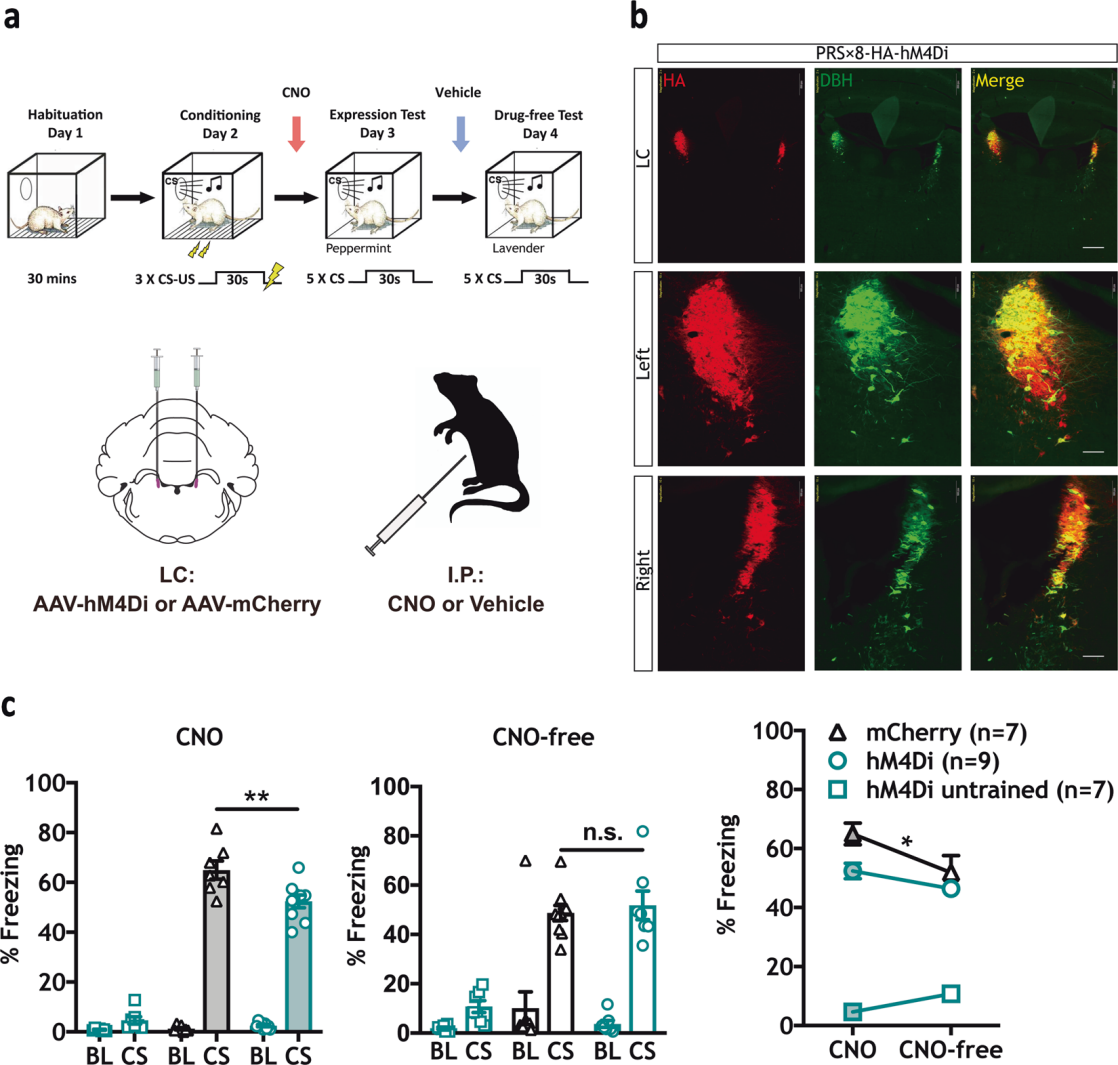
Chemogenetic inhibition of LC-NE signaling decreases CS-elicited freezing in threat-conditioned animals. **a** Top: Timeline indicating habituation (Day 1), conditioning (0.6 mA US) (Day 2), expression test (Day 3) and drug-free test (Day 4) phases. Bottom: Schematic depicting hM4Di or mCherry virus injection and CNO treatment strategy. **b** Representative IHC images show robust and selective targeting of hemagglutinin-tagged (HA) hM4Di receptors to DBH^+^ LC neurons. (Red = HA; Green = dopamine β hydroxylase (DBH); Yellow = indicates co-localization. Scale bars: top three panels = 500 µm, middle and bottom six panels = 100 µm. **c** On conditioning day, hM4Di paired (*n* = 9) and mCherry paired groups (*n* = 7) were threat conditioned, and an unpaired hM4Di control group (*n* = 7) received three tones alone. CNO (5.0 mg/kg) inhibition of LC-NE neurons significantly decreased CS-elicited freezing in trained hM4Di animals compared with mCherry controls (one-way ANOVA, *F* (2,20) = 126.4, ****P < 0.0001; Tukey’s MCS, **P < 0.01), with no difference observed between hM4Di paired and mCherry paired groups during the drug-free test. Within subject analysis revealed a slight reduction in CS-elicited freezing between days in the mCherry group, with no significant reduction in the hM4Di paired group (two-way RM ANOVA test, Interaction: *F* (2, 20) = 4.236, *p = 0.0292; Training x virus: *F* (2, 20) = 134.5, ****p < 0.0001; Time (CNO versus CNO-free): *F* (1,20) = 2.692, *p* = 0.1165; Sidak MCS, CNO versus CNO-free: hM4Di untrained, *p* = n.s., hM4Di trained, *p* = n.s., mCherry *p < 0.05). All error bars indicate mean ± SEM. *p < 0.05, **p < 0.01, ****p < 0.0001

LC neurons send NE efferents throughout the brain, including the amygdala [34–37]. We therefore tested whether LC-NE activity modulates the expression of defensive responses. Using AAV vectors expressing DREADDs (hM4Di) or a fluorescent reporter (mCherry) under the control of a synthetic promoter (PRS × 8) [21, 39, 44], we observed expression restricted to NE (dopamine β hydroxylase (DBH)-positive)) neurons in LC (Fig. 2b). Following bilateral AAV injections in LC, an hM4Di group (*n* = 9) and an mCherry (*n* = 7) group were trained using a moderate protocol (3 CS-US pairings, 0.6 mA US), while a third hM4Di group (*n* = 7) received three CS presentations without footshock to control for non-specific effects on freezing behavior. All animals received systemic injections of CNO (5.0 mg/kg) prior to the expression test (Fig. 2a). CNO significantly attenuated CS-elicited freezing levels only in conditioned animals expressing hM4Di compared to conditioned animals expressing mCherry alone (CS freezing: One-way ANOVA F (2, 20) = 126.4, ****p < 0.0001, Tukey’s MCT: hM4Di untrained versus mCherry trained, ****p < 0.0001, hM4Di untrained versus hM4Di trained, ****p < 0.0001, mCherry trained versus hM4Di trained, **p < 0.01), with no effect observed in the untrained behavioral controls expressing hM4Di (Fig. 2c, left panel). No significant effect was observed between trained groups in freezing levels during a CNO-free test and freezing remained negligible in the untrained group (Fig. 2c, center panel). In a separate analysis, c-Fos expression showed a trend, but no significant reduction in HA-expressing DBH neurons following CNO expression and training (Supplementary Fig. 5a). These data suggest that LC-NE activity positively modulates the expression of learned threat reactions.

### Chemogenetic activation of LC enhances freezing to a conditioned cue

**Fig. 3:**
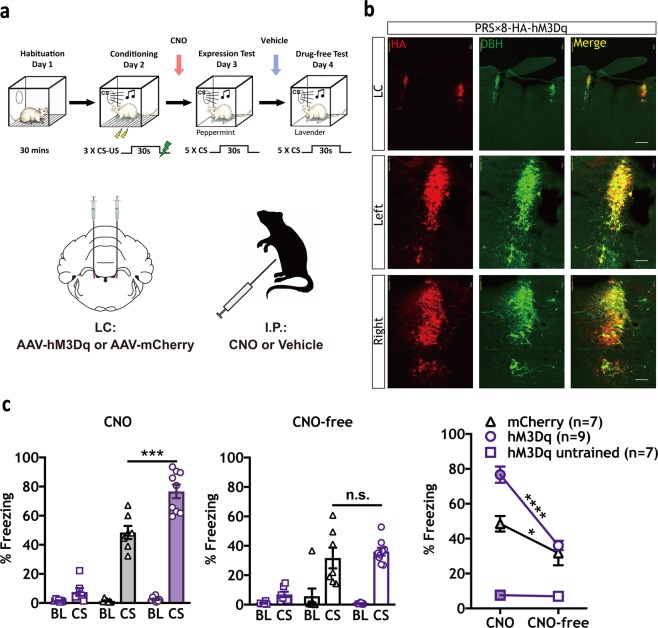
Chemogenetic activation of LC-NE signaling increases CS-elicited freezing in threat-conditioned animals. **a** Top: Timeline indicating habituation (Day 1), mild conditioning (0.4 mA US) (Day 2), expression test (Day 3), and drug-free test (Day 4) phases. Bottom: Schematic depicting hM3Dq or mCherry virus injection and CNO treatment strategy. **b** Representative IHC images show robust and selective targeting of hM3Dq-HA to DBH^+^ LC neurons. (Red = HA; Green = DBH; Yellow = co-localization). Scale bars: top three panels = 500 µm, middle and bottom six panels = 100 µm. **c** Systemic injection of CNO (1.0 mg/kg) prior to the expression test significantly enhanced freezing in the trained hM3Dq group (*n* = 9) compared with the trained mCherry group (*n* = 7; left panel, one-way ANOVA, *F* (2,20) = 69.54, ****P < 0.0001, Tukey’s MCS, ***P < 0.001). No differences were observed between groups during a CNO-free test (center panel). A difference in CS-elicited freezing was observed in trained animals between CNO- and CNO-free tests (two-way RM ANOVA test, Interaction: *F* (2, 20) = 16.62, ****p < 0.0001; Training X virus: *F* (2, 20) = 55.64, ****p < 0.0001, Time (CNO versus CNO-free): *F* (1,20) = 43.45, ****p < 0.0001; Sidak MCS, CNO versus CNO-free: hM4Di untrained, *p* = n.s., hM3Dq trained, ****p < 0.0001, mCherry trained, *p < 0.05). All error bars indicate mean ± SEM. *p < 0.05, ***p < 0.001, ****p < 0.0001

Next, excitatory DREADDs (hM3Dq) were used to determine how stimulation of LC-NE activity would affect CS-elicited freezing. hM3Dq- and mCherry-immunopositive neurons were observed to co-localize with the DBH-positive neurons in LC [21, 39], red fluorescence was detected throughout the entire LC but not in neighboring regions (Fig. 3b, Supplementary Fig. 2), which showed that viral targeting was LC specific. An hM3Dq group (*n* = 9) and an mCherry group (*n* = 7) were trained using a weak protocol (to avoid ceiling effects, three CS-US pairings, 0.4 mA shock), and a third hM3Dq group (*n* = 7) received three CS-alone presentations without footshock to control for unconditioned freezing behaviors. Systemic CNO (1.0 mg/kg) significantly increased CS-elicited freezing levels in trained hM3Dq-expressing animals compared with mCherry-expressing animals (*F* (2, 20) = 69.54, ****p < 0.0001, Tukey’s MCT: hM3Dq untrained versus mCherry trained, ****p < 0.0001, hM3Dq untrained versus hM3Dq trained, ****p < 0.0001, mCherry trained versus hM3Dq trained, ***p < 0.001), with minimal CS-elicited freezing responses observed in the untrained control group (Fig. 3c, left panel). No significant effects were observed between trained groups during a CNO-free test (Fig. 3c, center panel). In a separate group of animals, c-Fos expression in HA-expressing DBH neurons was significantly increased by CNO (*p < 0.05; Supplementary Fig. 5b). Taken together, these and the hM4Di data (Fig. 2) suggest that LC activity positively modulates CS-elicited freezing.

### Pharmacological blockade of β-ARs in CeA attenuates freezing

**Fig. 4:**
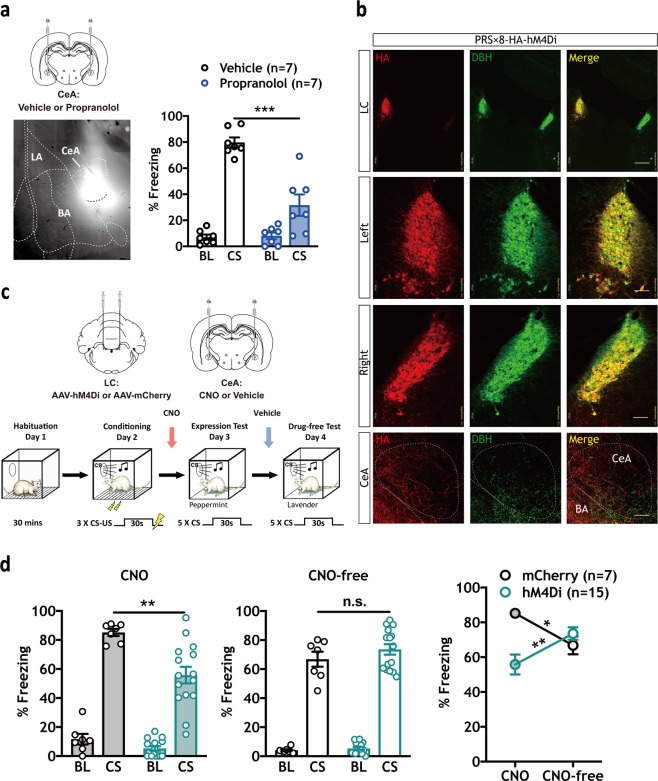
CeA blockade of β-ARs or chemogenetic inhibition of LC-NE axon terminals in the CeA decreases CS-elicited freezing. **a** Local propranolol infusions in CeA (1.0 µg/0.3 µl/side) significantly reduced CS-elicited freezing (***p = 0.0002) in expression test as described above. A fluorophore-conjugated propranolol was infused to confirm the spread of the drug in CeA. **b** Robust and selective targeting of hM4Di-HA to DBH^+^ LC neurons and detectable expression in axons projecting to CeA after six weeks (Red = HA; Green = DBH; Yellow = co-localization). Scale bars: top three LC panels = 500 µm, middle six LC panels = 100 µm, bottom three CeA panels = 50 µm. **c** Virus and CNO infusion strategy and experimental timeline. **d** CNO infusions in CeA (1.0 mM/0.3 µl/side) significantly reduced CS-elicited freezing in hM4Di animals (*n* = 15) compared with the mCherry (*n* = 7) group, Mann–Whitney *U*, **p = 0.0015). No differences were observed between groups during the CNO-free test (center panel). Differences were observed in CS-elicited freezing in both groups between CNO- and CNO-free tests (two-way RM ANOVA test, Interaction: *F* (1, 20) = 20.13, ***p = 0.0002; Virus: *F* (1, 20) = 3.088, *p* = 0.0942; Time (CNO versus CNO-free): *F* (1,20) = 0.005266, *p* = 0.9429; Sidak MCS, CNO versus CNO-free: mCherry, *p < 0.05, hM4Di, **p < 0.01). All error bars indicate mean ± SEM. *p < 0.05, **p < 0.01, ***p < 0.001. Central amygdala (CeA), lateral amygdala (LA) basal amygdala (BA)

We found that pharmacological and chemogenetic manipulations of brain NE activity bidirectionally modulated behavioral expression. We next tested the hypothesis that NE activity in the CeA positively modulates CS-elicited freezing. Rats received bilateral microinjections of the β-AR antagonist propranolol or vehicle in the CeA prior to the expression test. Results show that propranolol (1.0 µg/0.3 µL/side) significantly attenuated CS-elicited freezing levels compared with vehicle controls (*n* = 7/group, *t* (12) = 5.324, ***p = 0.0002) (Fig. 4a, right panel). Therefore, β-AR activity specifically in CeA mediates the expression of responses to learned threats.

### Chemogenetic inhibition of LC terminals in CeA attenuates freezing

We have shown that NE neurons in LC and β-AR activity in CeA play a critical role in defensive responses. To test the LC→CeA circuit, we combined targeted expression of hM4Di in LC-NE with direct CNO infusions to block axon terminal activity in CeA (Fig. 4b). Six weeks following surgery, animals were trained using a standard conditioning protocol (Fig. 4c) and memory tested the subsequent day. Pre-test infusion of CNO in CeA (1.0 mM/0.3 µl/side) in hM4Di-expressing animals (*n* = 15) significantly attenuated freezing levels compared with mCherry controls (Fig. 4d left panel, *n* = 7, Mann–Whitney *U* test, **p = 0.0015. During a CNO-free test, intra-CeA infusion of vehicle did not significantly affect freezing behavior between groups (Fig. 4d, center panel). To control for drug spread affecting the adjacent BLA, some animals were infused with CNO directly in BLA (Supplementary Fig. 5c). Consistent with previous reports [1, 45], no effect was observed on behavioral expression. These data support the hypothesis that LC→CeA, but not LC→BLA circuit activity is necessary for the expression of conditioned threat reactions.

### Chemogenetic activation of LC-NE terminals in CeA enhances CS-elicited freezing

**Fig. 5:**
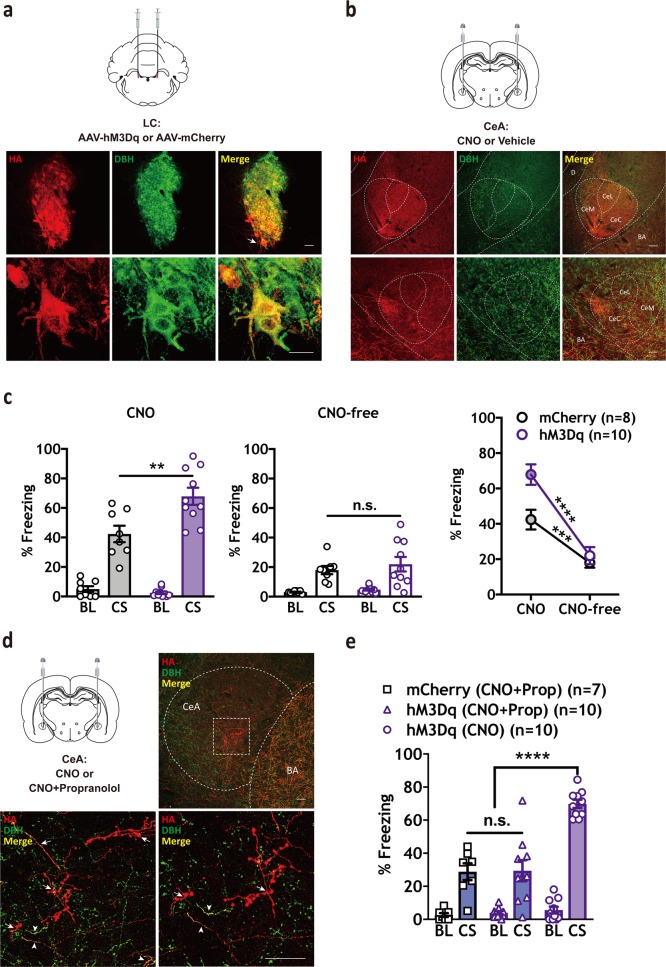
Chemogenetic activation of LC-NE terminals in CeA increases CS-elicited freezing behavior and is blocked by β-AR antagonist. **a** hM3Dq (HA) expression in LC (Red = HA; Green = DBH; Yellow = co-localization). Arrow indicates the magnification of the same LC neuron. Scale bars: LC panels = 50 µm. **b** HA-immunopositive terminals from LC were detected in CeA. Scale bars: CeA panels = 50 µm. **c** Infusions of CNO (1.0 mM/0.3 µl/side) led to a significant increase in CS-elicited freezing in hM3Dq (*n* = 10) animals compared with mCherry controls (left panel, *n* = 8, t (16) = 3.114, **p = 0.0067), with no difference observed between groups during a CNO-free test (center panel). Differences were observed in CS-elicited freezing in both groups between CNO- and CNO-free tests (two-way RM ANOVA test, Interaction: *F* (1, 16) = 11.78, **p = 0.0034; Virus: *F* (1, 16) = 5.197, *p = 0.0367; Time (CNO versus CNO-free): *F* (1,16) = 125.7, ****p < 0.0001; Sidak MCS, CNO versus CNO-free: mCherry, ***p < 0.001, hM3Dq, ****p < 0.0001). **d** Prior to the expression test, animals received intra-CeA infusions of CNO alone (hM3Dq (CNO); (*n* = 10)), or a cocktail of CNO and propranolol (1.0 mM/0.3 µl/side + 1.0 µg/0.3 µl/side) in hM3Dq (*n* = 10) and mCherry (*n* = 7) animals. Representative IHC images show robust and selective targeting of hM3Dq-HA to DBH^+^ LC neurons and strong expression in CeA terminals. (Red = HA; Green = DBH; Yellow = overlapping indicates co-localization). Arrow indicates the magnification of the LC-NE terminals in CeA. Scale bars, CeA panels = 50 µm. **e** Propranolol significantly reduced the effect of CNO in hM3Dq-expressing animals (one-way ANOVA, *F* (2, 24) = 23.77, ****P < 0.0001, Tukey’s MCS, mCherry (CNO + Prop) versus hM3Dq (CNO), ****p < 0.0001 and hM3Dq (CNO + Prop) versus hM3Dq (CNO), ****p < 0.0001). All error bars indicate mean ± SEM. **p < 0.01, ****p < 0.0001. Central medial amygdala (CeM), Central lateral amygdala (CeL), Central capsular amygdala (Cec), Basal amygdala (BA), Central amygdala (CeA)

To further confirm a role for a LC→CeA circuit in defensive responses, we combined targeted expression of hM3Dq in LC-NE with direct CNO infusions to activate axon terminals in CeA. Six weeks following surgery, animals were trained using a weak conditioning protocol followed by an expression test the next day (Fig. 5c). Following bilateral microinjections of CNO in CeA (1.0 mM/0.3 µl/side), we found that CNO significantly increased freezing levels in hM3Dq animals compared with the mCherry control group (Fig. 5c, left panel, *n* = 8–10/group, *t* (16) = 3.114, **p = 0.0067). In a subsequent CNO-free test, CS-elicited freezing was not different between groups (Fig. 5c, center panel). To control for CNO effects on adjacent regions, some animals received CNO in BLA (Supplementary Fig. 5d). As with the BLA infusion control in hM4Di-expressing animals, no effect was observed on CS-elicited freezing behavior.

### Enhancement of CS-elicited freezing by chemogenetic activation of the LC→CeA circuit requires β-AR activity in CeA

Next, we determined if chemogenetic enhancement of CS-elicited freezing requires NE activity at β-ARs in CeA. Prior to the expression test, hM3Dq rats were administered bilateral microinjections of either CNO alone (1.0 mM/0.3 µl/side) or a cocktail of propranolol (1.0 µg/0.3 µl/side) and CNO (1.0 mM/0.3 µl/side) in CeA. An mCherry group was also administered the propranolol-CNO cocktail. Results showed that CNO alone significantly enhanced CS-elicited freezing in hM3Dq animals, whereas inhibition of β-ARs in CeA significantly reduced this effect (one-way ANOVA: *F* (2, 24) = 23.77, ****p < 0.0001, Tukey’s MCS: mCherry (CNO + Prop) versus hM3Dq (CNO + Prop), n.s., mCherry (CNO + Prop) versus hM3Dq (CNO), ****p < 0.0001, hM3Dq (CNO + Prop) versus hM3Dq (CNO), ****p < 0.0001) (Fig. 5e, *n* = 7–10/group). No difference was observed between hM3Dq (CNO + Prop) and mCherry (CNO + Prop) groups. These data suggest that freezing reactions require NE release from LC terminals in CeA.

## Discussion

Here we establish the neural underpinnings of noradrenergic modulation of Pavlovian defensive reactions. First, we found that propranolol-mediated blockade of β-ARs significantly reduced behavioral expression of PTC [43] (Fig. 1b, Supplementary Fig. 4b, 4d), whereas systemic activation of β_2_-ARs using procaterol enhanced it (Fig. 1c). Chemogenetic reduction (Fig. 2c) or enhancement (Fig. 3c) of noradrenergic LC activity confirmed the role of endogenous noradrenergic activity in behavioral expression. Furthermore, blockade of β-ARs in the CeA, the major output nucleus of the amygdala mediating defensive reactions (Fig. 4a) or inhibition of the LC→CeA circuit (Fig. 4d) yielded results consistent with systemic propranolol (Fig. 1b) and chemogenetic LC inhibition (Fig. 2c). As with procaterol injections (Fig. 1c) and LC activation (Fig. 3c), stimulation of LC→CeA inputs enhanced CS-elicited freezing (Fig. 5c). This enhancement was blocked by direct infusion of propranolol, suggesting that NE released from LC axons acts through β-ARs in CeA (Fig. 5e). Consistent with previous work [1, 45], our control studies (Supplementary Fig. 5c, 5d) found no effect of BLA DREADD manipulations on the expression of defensive responses, suggesting that other targets of the NE system, e.g., CeA are responsible. Taken together, these data suggest that LC→CeA axonal projections release NE into the CeA where β-AR activation positively modulates the degree of CS-elicited freezing.

Noradrenergic LC neurons phasically respond to salient stimuli (learned or novel) in all sensory modalities [46, 47]. NE may improve selectivity or increase the magnitude of neuronal responses to sensory stimulation [39] and promote synaptic transmission and plasticity in threat processing circuits of the amygdala [48]. Indeed, NE signaling through β-ARs in LA and/or BLA can regulate the acquisition [1, 2, 28], consolidation [2, 31], reconsolidation [49, 50], and extinction [51, 52] of memory. However, neither propranolol nor DREADD-mediated inhibition or activation at axon terminals in BLA during an expression test affected CS-elicited freezing [1, 45] (also see Supplementary Fig. 5c, 5d).

A recent study showed that sustained (10–15 s) high-frequency (10 Hz) photostimulation of LC can lead to reversible ‘behavioral arrest’ [20]. However, this strong stimulation decreased cortical release of NE, suggesting that brain-wide depletion of NE may be responsible for the observed effects on behavior. On the other hand, DREADD-mediated LC excitation, which we used here (Fig. 3), was shown to tonically increase LC neuronal firing at physiological frequencies (~5 Hz) [39]. Indeed, using this same viral vector, Kane et al. did not observe behavioral arrest, but did find a disruption in a foraging behavior task [53], possibly due to ‘decision noise’ created by persistent LC activity. This is perhaps consistent with our previous study showing that the invigorating effects of a CS on an avoidance task (Pavlovian-to-instrumental transfer, PIT) were disrupted by hM3Dq-mediated excitation of LC [54]. Notably, baseline shuttling behavior (before the CS) was not affected, suggesting that the PIT deficit was due to an inability of the CS to motivate responding. Taken together, these results suggest that conditioned aversive arousal modulates both Pavlovian and instrumental responses and are consistent with the theory that phasic activation of NE neurons of the LC, and NE release in CeA, could permit rapid behavioral adaptation to changing environmental imperatives [21, 55, 56].

Studies using other aversive learning paradigms such as inhibitory avoidance also find an important role for NE activity in amygdala, although there are discrepancies when compared with PTC [30–32, 52]. For example, NE signaling in the LA and/or BLA is required for memory acquisition in PTC but not for inhibitory avoidance [1, 2, 30–32, 52]. These differences suggest that distinct behavioral and neural processes may underlie each task. Indeed, inhibitory avoidance involves contextual and instrumental learning that are not required for cued PTC [2, 30–32, 52]. Moreover, a recent study showed that low, tonic optogenetic stimulation (5 Hz for 20 min) of LC-NE neurons innervating the BLA can increase anxiety-like behaviors and conditioned aversion, the latter of which shares similarities to inhibitory avoidance [33]. In contrast to LA/BLA, very little is known about NE activity in CeA in both inhibitory avoidance and PTC. A single study looking at NE signaling in CeA found no role in the consolidation of inhibitory avoidance memory but did not test for expression effects [31].

While the NE LC→CeA circuit modulated conditioned memory expression, it did not affect unconditioned freezing (Figs. 2c, 3c). Although pre-CS freezing was slightly reduced in two experiments (by systemic propranolol (Fig. 1b) and systemic injections of CNO in LC hM4Di animals (Fig. 2c)), CNO infusion in CeA in hM4Di animals did not change pre-CS freezing levels (Fig. 4d). Also, baseline freezing was not affected in any of the excitatory manipulations, including the potentiation of β_2_-AR activity with procaterol (Fig. 1c) or hM3Dq-mediated enhancement of LC (Fig. 3c) and LC→CeA activity (Fig. 5c, e). Changes in pre-CS freezing levels would be expected if brain manipulations of NE activity affected unconditioned freezing.

We used chemogenetic manipulations for many of our studies. There has been recent controversy over the use of CNO, which can be metabolically converted into the atypical antipsychotic clozapine [57]. We attempted to circumvent this caveat in several ways. First, we chose systemic doses of CNO that were low enough to minimize this effect [58, 59], and we used mCherry controls that received the same CNO treatments as experimental groups. Furthermore, we supplemented systemic CNO injection studies with direct, intracranial infusion of CNO to bypass the peripheral metabolism of CNO to clozapine.

Although LC is a major source of NE to the forebrain [60–62], NE cell groups in the medulla and subcoeruleus region also send strong projections throughout the brain [63]. Much existing evidence shows A2 adrenergic neurons (located in the dorsal vagal complex, including the nucleus of the solitary tract (NTS)), also project to CeA [6, 63–65]. Our chemogenetic inhibitory studies with hM4Di show subtle but significant effects, which may suggest that other sources of NE, e.g., A2, summate with LC-NE in the CeA to positively modulate CS-elicited freezing. Indeed, one study showed that A2 neurons are activated by threat-conditioned stimuli [66], and a more recent study shows that neurons from the NTS can negatively regulate anxiety-like behavior [6]. It is also possible that DREADD-mediated neuromodulatory inhibition is not sufficient to fully shunt excitatory responses in NE cells (see Supplementary Fig. 5a). Future work delineating the relative contribution of other NE inputs to defensive responses is therefore warranted.

Given the canonical role of the CeA in expression, in the current studies we focused on this particular phase of conditioning. However, there is some evidence that the CeA itself can mediate Pavlovian memory formation (memory acquisition and/or consolidation) [13, 14, 67, 68]. Recent work exploring the mechanisms underlying this phenomenon suggests that the CeA may convey information about the unconditioned stimulus to the LA during learning [67]. Further studies are needed to determine if NE modulates CeA during the acquisition phase.

Our studies describe the LC→CeA projection, but a reciprocal CeA→LC projection has also been reported [69]. This circuit is modulated by corticotrophin releasing factor (CRF), which is enhanced by stress and may represent a feedforward excitatory mechanism for the LC-NE-mediated responses to threat. If and how this reciprocal circuit is involved in defensive reactions is an important subject of future research.

Symptoms of fear and anxiety disorders involve exaggerated responses to stimuli, whether or not it is a threatening [70], and studies in humans and animals suggest this may be due to dysfunction of the amygdala, the LC [71] and NE activity in amygdala [9, 10, 24, 72–77]. Together with previous studies, our findings suggested the systemic propranolol treatment can temper the expression of defensive responses [42]. Indeed, this models the efficacy of propranolol to quell the symptoms of stage fright or memory retrieval in PTSD patients [24]. Notably, we do not replicate previous findings showing that systemic propranolol given during the expression test can impair reconsolidation [49, 50] as determined by CS-elicited freezing levels in a second expression test. However, as has been highlighted by others [24], the strength of training may determine the efficacy of this manipulation on reconsolidation. Indeed, for our experiments we used three CS–US pairings as opposed to one pairing used in these other studies [49, 50].

The current findings suggest a mechanism for emotional regulation in health and disease. Specifically, the LC→CeA circuit may underlie exaggerated reactions to stimuli and may explain the efficacy of β-AR antagonists like propranolol in fear and anxiety disorders [78–80].

## Supplementary information

Supplementary Information

## References

[1] Bush DE, Caparosa EM, Gekker A, Ledoux J (2010). Beta-adrenergic receptors in the lateral nucleus of the amygdala contribute to the acquisition but not the consolidation of auditory fear conditioning. Front Behav Neurosci.

[2] Schiff HC, Johansen JP, Hou M, Bush DE, Smith EK, Klein JE (2017). Beta-adrenergic receptors regulate the acquisition and consolidation phases of aversive memory formation through distinct, temporally regulated signaling pathways. Neuropsychopharmacology.

[3] Keifer OP, Hurt RC, Ressler KJ, Marvar PJ (2015). The physiology of fear: reconceptualizing the role of the central amygdala in fear learning. Physiology.

[4] Fadok JP, Markovic M, Tovote P, Luthi A (2018). New perspectives on central amygdala function. Curr Opin Neurobiol.

[5] Li B (2019). Central amygdala cells for learning and expressing aversive emotional memories. Curr Opin Behav Sci.

[6] Chen YW, Das M, Oyarzabal EA, Cheng Q, Plummer NW, Smith KG (2019). Genetic identification of a population of noradrenergic neurons implicated in attenuation of stress-related responses. Mol Psychiatry.

[7] LeDoux JE (2014). Coming to terms with fear. PNAS.

[8] Herry C, Johansen JP (2014). Encoding of fear learning and memory in distributed neuronal circuits. Nat Neurosci.

[9] Davis M (1992). The role of the amygdala in fear and anxiety. Annu Rev Neurosci.

[10] Fenster RJ, Lebois LAM, Ressler KJ, Suh J (2018). Brain circuit dysfunction in post-traumatic stress disorder: from mouse to man. Nat Rev Neurosci.

[11] Han S, Soleiman MT, Soden ME, Zweifel LS, Palmiter RD (2015). Elucidating an affective pain circuit that creates a threat memory. Cell.

[12] Yu K, Garcia da Silva P, Albeanu DF, Li B (2016). Central amygdala somatostatin neurons gate passive and active defensive behaviors. J Neurosci.

[13] Wilensky AE, Schafe GE, Kristensen MP, LeDoux JE (2006). Rethinking the fear circuit: the central nucleus of the amygdala is required for the acquisition, consolidation, and expression of Pavlovian fear conditioning. J Neurosci.

[14] Li H, Penzo MA, Taniguchi H, Kopec CD, Huang ZJ, Li B (2013). Experience-dependent modification of a central amygdala fear circuit. Nat Neurosci.

[15] Pitkanen A, Savander V, LeDoux JE (1997). Organization of intra-amygdaloid circuitries in the rat: an emerging framework for understanding functions of the amygdala. Trends Neurosci.

[16] Tovote P, Esposito MS, Botta P, Chaudun F, Fadok JP, Markovic M (2016). Midbrain circuits for defensive behaviour. Nature.

[17] Ciocchi S, Herry C, Grenier F, Wolff SB, Letzkus JJ, Vlachos I (2010). Encoding of conditioned fear in central amygdala inhibitory circuits. Nature.

[18] LeDoux JE, Iwata J, Cicchetti P, Reis DJ (1988). Different projections of the central amygdaloid nucleus mediate autonomic and behavioral correlates of conditioned fear. J Neurosci.

[19] Ozawa T, Ycu EA, Kumar A, Yeh LF, Ahmed T, Koivumaa J (2017). A feedback neural circuit for calibrating aversive memory strength. Nat Neurosci.

[20] Carter ME, Yizhar O, Chikahisa S, Nguyen H, Adamantidis A, Nishino S (2010). Tuning arousal with optogenetic modulation of locus coeruleus neurons. Nat Neurosci.

[21] Vazey EM, Moorman DE, Aston-Jones G (2018). Phasic locus coeruleus activity regulates cortical encoding of salience information. PNAS.

[22] Saha S, Drinkhill MJ, Moore JP, Batten TF (2005). Central nucleus of amygdala projections to rostral ventrolateral medulla neurones activated by decreased blood pressure. Eur J Neurosci.

[23] Rodrigues SM, LeDoux JE, Sapolsky RM (2009). The influence of stress hormones on fear circuitry. Annu Rev Neurosci.

[24] Giustino TF, Maren S (2018). Noradrenergic modulation of fear conditioning and extinction. Front Behav Neurosci.

[25] Chen FJ, Sara SJ (2007). Locus coeruleus activation by foot shock or electrical stimulation inhibits amygdala neurons. Neuroscience.

[26] Uematsu A, Tan BZ, Ycu EA, Cuevas JS, Koivumaa J, Junyent F (2017). Modular organization of the brainstem noradrenaline system coordinates opposing learning states. Nat Neurosci.

[27] McCall JG, Al-Hasani R, Siuda ER, Hong DY, Norris AJ, Ford CP (2015). CRH engagement of the locus coeruleus noradrenergic system mediates stress-induced anxiety. Neuron.

[28] Johansen JP, Diaz-Mataix L, Hamanaka H, Ozawa T, Ycu E, Koivumaa J (2014). Hebbian and neuromodulatory mechanisms interact to trigger associative memory formation. PNAS.

[29] Buffalari DM, Grace AA (2007). Noradrenergic modulation of basolateral amygdala neuronal activity: opposing influences of alpha-2 and beta receptor activation. J Neurosci.

[30] Ferry B, McGaugh JL (2000). Role of amygdala norepinephrine in mediating stress hormone regulation of memory storage. Acta Pharm Sin.

[31] Quirarte GL, Galvez R, Roozendaal B, McGaugh JL (1998). Norepinephrine release in the amygdala in response to footshock and opioid peptidergic drugs. Brain Res.

[32] Cahill L, Prins B, Weber M, McGaugh JL (1994). Beta-adrenergic activation and memory for emotional events. Nature.

[33] McCall JG, Siuda ER, Bhatti DL, Lawson LA, McElligott ZA, Stuber GD et al. Locus coeruleus to basolateral amygdala noradrenergic projections promote anxiety-like behavior. Elife 2017;6:e18247. 10.7554/eLife.18247 PMC5550275 28708061

[34] Jones BE, Yang TZ (1985). The efferent projections from the reticular formation and the locus coeruleus studied by anterograde and retrograde axonal transport in the rat. J Comp Neurol.

[35] Moore RY, Bloom FE (1979). Central catecholamine neuron systems: anatomy and physiology of the norepinephrine and epinephrine systems. Annu Rev Neurosci.

[36] Fallon JH, Koziell DA, Moore RY (1978). Catecholamine innervation of the basal forebrain. II. Amygdala, suprarhinal cortex and entorhinal cortex. J Comp Neurol.

[37] Uematsu A, Tan BZ, Johansen JP (2015). Projection specificity in heterogeneous locus coeruleus cell populations: implications for learning and memory. Learn Mem.

[38] Armbruster BN, Li X, Pausch MH, Herlitze S, Roth BL (2007). Evolving the lock to fit the key to create a family of G protein-coupled receptors potently activated by an inert ligand. PNAS.

[39] Vazey EM, Aston-Jones G (2014). Designer receptor manipulations reveal a role of the locus coeruleus noradrenergic system in isoflurane general anesthesia. PNAS.

[40] Paxinos George, Watson Charles (1982). WITHDRAWN: Acknowledgements. The Rat Brain in Stereotaxic Coordinates.

[41] Mahler SV, Vazey EM, Beckley JT, Keistler CR, McGlinchey EM, Kaufling J (2014). Designer receptors show role for ventral pallidum input to ventral tegmental area in cocaine seeking. Nat Neurosci.

[42] McAllister, McAllister, Douglass. (1971). Inverse relationship between shock intensity and shuttle-box avoidance learning in rats—reinforcement explanation. J Comp Physiol Psychol.

[43] Rodriguez-Romaguera J, Sotres-Bayon F, Mueller D, Quirk GJ (2009). Systemic propranolol acts centrally to reduce conditioned fear in rats without impairing extinction. Biol Psychiatry.

[44] Hwang DY, Carlezon WA, Isacson O, Kim KS (2001). A high-efficiency synthetic promoter that drives transgene expression selectively in noradrenergic neurons. Hum Gene Ther.

[45] Vetere G, Piserchia V, Borreca A, Novembre G, Aceti M, Ammassari-Teule M (2013). Reactivating fear memory under propranolol resets pre-trauma levels of dendritic spines in basolateral amygdala but not dorsal hippocampus neurons. Front Behav Neurosci.

[46] Aston-Jones G, Bloom FE (1981). Norepinephrine-containing locus coeruleus neurons in behaving rats exhibit pronounced responses to non-noxious environmental stimuli. J Neurosci.

[47] Foote SL, Aston-Jones G, Bloom FE (1980). Impulse activity of locus coeruleus neurons in awake rats and monkeys is a function of sensory stimulation and arousal. PNAS.

[48] Tully K, Li Y, Tsvetkov E, Bolshakov VY (2007). Norepinephrine enables the induction of associative long-term potentiation at thalamo-amygdala synapses. PNAS.

[49] Debiec J, Ledoux JE (2004). Disruption of reconsolidation but not consolidation of auditory fear conditioning by noradrenergic blockade in the amygdala. Neuroscience.

[50] Debiec J, LeDoux JE (2006). Noradrenergic signaling in the amygdala contributes to the reconsolidation of fear memory: treatment implications for PTSD. Ann NY Acad Sci.

[51] Debiec J, Bush DE, LeDoux JE (2011). Noradrenergic enhancement of reconsolidation in the amygdala impairs extinction of conditioned fear in rats—a possible mechanism for the persistence of traumatic memories in PTSD. Depression Anxiety.

[52] Berlau DJ, McGaugh JL (2006). Enhancement of extinction memory consolidation: the role of the noradrenergic and GABAergic systems within the basolateral amygdala. Neurobiol Learn Mem.

[53] Kane GA, Vazey EM, Wilson RC, Shenhav A, Daw ND, Aston-Jones G (2017). Increased locus coeruleus tonic activity causes disengagement from a patch-foraging task. Cogn, Affect Behav Neurosci.

[54] Campese Vincent D., Soroeta Jose M., Vazey Elena M., Aston-Jones Gary, LeDoux Joseph E., Sears Robert M. (2017). Noradrenergic Regulation of Central Amygdala in Aversive Pavlovian-to-Instrumental Transfer. eneuro.

[55] Bouret S, Sara SJ (2005). Network reset: a simplified overarching theory of locus coeruleus noradrenaline function. Trends Neurosci.

[56] Aston-Jones G, Waterhouse B (2016). Locus coeruleus: from global projection system to adaptive regulation of behavior. Brain Res.

[57] Gomez JL, Bonaventura J, Lesniak W, Mathews WB, Sysa-Shah P, Rodriguez LA (2017). Chemogenetics revealed: DREADD occupancy and activation via converted clozapine. Science.

[58] MacLaren Duncan A. A., Browne Richard W., Shaw Jessica K., Krishnan Radhakrishnan Sandhya, Khare Prachi, España Rodrigo A., Clark Stewart D. (2016). Clozapine N-Oxide Administration Produces Behavioral Effects in Long–Evans Rats: Implications for Designing DREADD Experiments. eneuro.

[59] Mahler Stephen V, Aston-Jones Gary (2018). CNO Evil? Considerations for the Use of DREADDs in Behavioral Neuroscience. Neuropsychopharmacology.

[60] Jones BE, Moore RY (1977). Ascending projections of the locus coeruleus in the rat. II. Brain Res.

[61] Aston-Jones G, Cohen JD (2005). An integrative theory of locus coeruleus-norepinephrine function: adaptive gain and optimal performance. Annu Rev Neurosci.

[62] Sara SJ (2009). The locus coeruleus and noradrenergic modulation of cognition. Nat Rev Neurosci.

[63] Rinaman L (2011). Hindbrain noradrenergic A2 neurons: diverse roles in autonomic, endocrine, cognitive, and behavioral functions. Am J Physiol Regulatory, Integr Comp Physiol.

[64] Robertson SD, Plummer NW, de Marchena J, Jensen P (2013). Developmental origins of central norepinephrine neuron diversity. Nat Neurosci.

[65] Saha S, Henderson Z, Batten TF (2002). Somatostatin immunoreactivity in axon terminals in rat nucleus tractus solitarii arising from central nucleus of amygdala: coexistence with GABA and postsynaptic expression of sst2A receptor. J Chem Neuroanat.

[66] Zhu L, Onaka T (2002). Involvement of medullary A2 noradrenergic neurons in the activation of oxytocin neurons after conditioned fear stimuli. Eur J Neurosci.

[67] Yu K, Ahrens S, Zhang X, Schiff H, Ramakrishnan C, Fenno L (2017). The central amygdala controls learning in the lateral amygdala. Nat Neurosci.

[68] Goosens KA, Maren S (2003). Pretraining NMDA receptor blockade in the basolateral complex, but not the central nucleus, of the amygdala prevents savings of conditional fear. Behav Neurosci.

[69] Valentino RJ, Van Bockstaele E (2008). Convergent regulation of locus coeruleus activity as an adaptive response to stress. Eur J Pharm.

[70] Parsons RG, Ressler KJ (2013). Implications of memory modulation for post-traumatic stress and fear disorders. Nat Neurosci.

[71] Naegeli C, Zeffiro T, Piccirelli M, Jaillard A, Weilenmann A, Hassanpour K (2018). Locus coeruleus activity mediates hyperresponsiveness in posttraumatic stress disorder. Biol Psychiatry.

[72] Ronzoni G, Del Arco A, Mora F, Segovia G (2016). Enhanced noradrenergic activity in the amygdala contributes to hyperarousal in an animal model of PTSD. Psychoneuroendocrinology.

[73] Bremner JD, Krystal JH, Southwick SM, Charney DS (1996). Noradrenergic mechanisms in stress and anxiety: I. preclinical studies. Synapse.

[74] Southwick SM, Krystal JH, Bremner JD, Morgan CA, Nicolaou AL, Nagy LM (1997). Noradrenergic and serotonergic function in posttraumatic stress disorder. Arch Gen psychiatry.

[75] Geracioti TD, Baker DG, Ekhator NN, West SA, Hill KK, Bruce AB (2001). CSF norepinephrine concentrations in posttraumatic stress disorder. Am J Psychiatry.

[76] Giustino TF, Fitzgerald PJ, Maren S (2016). Revisiting propranolol and PTSD: memory erasure or extinction enhancement?. Neurobiol Learn Mem.

[77] Diaz-Mataix L, Piper WT, Schiff HC, Roberts CH, Campese VD, Sears RM (2017). Characterization of the amplificatory effect of norepinephrine in the acquisition of Pavlovian threat associations. Learn Mem.

[78] Pitman RK, Sanders KM, Zusman RM, Healy AR, Cheema F, Lasko NB (2002). Pilot study of secondary prevention of posttraumatic stress disorder with propranolol. Biol Psychiatry.

[79] Hurlemann R, Walter H, Rehme AK, Kukolja J, Santoro SC, Schmidt C (2010). Human amygdala reactivity is diminished by the beta-noradrenergic antagonist propranolol. Psychol Med.

[80] Cain CK, Maynard GD, Kehne JH (2012). Targeting memory processes with drugs to prevent or cure PTSD. Exp Opin Investig Drugs.

