# Supplementary material for: A brainstem-central amygdala circuit underlies defensive responses to learned threats
Source: Mol Psychiatry. 2019 Nov 22;25(3):640–54. doi: 10.1038/s41380-019-0599-6 (PMC7042728; doi:10.1038/s41380-019-0599-6)
Supplement: Supplementary file 1 — Supplementary Information [file 41380_2019_599_MOESM1_ESM.pdf]

## Supplementary Figures

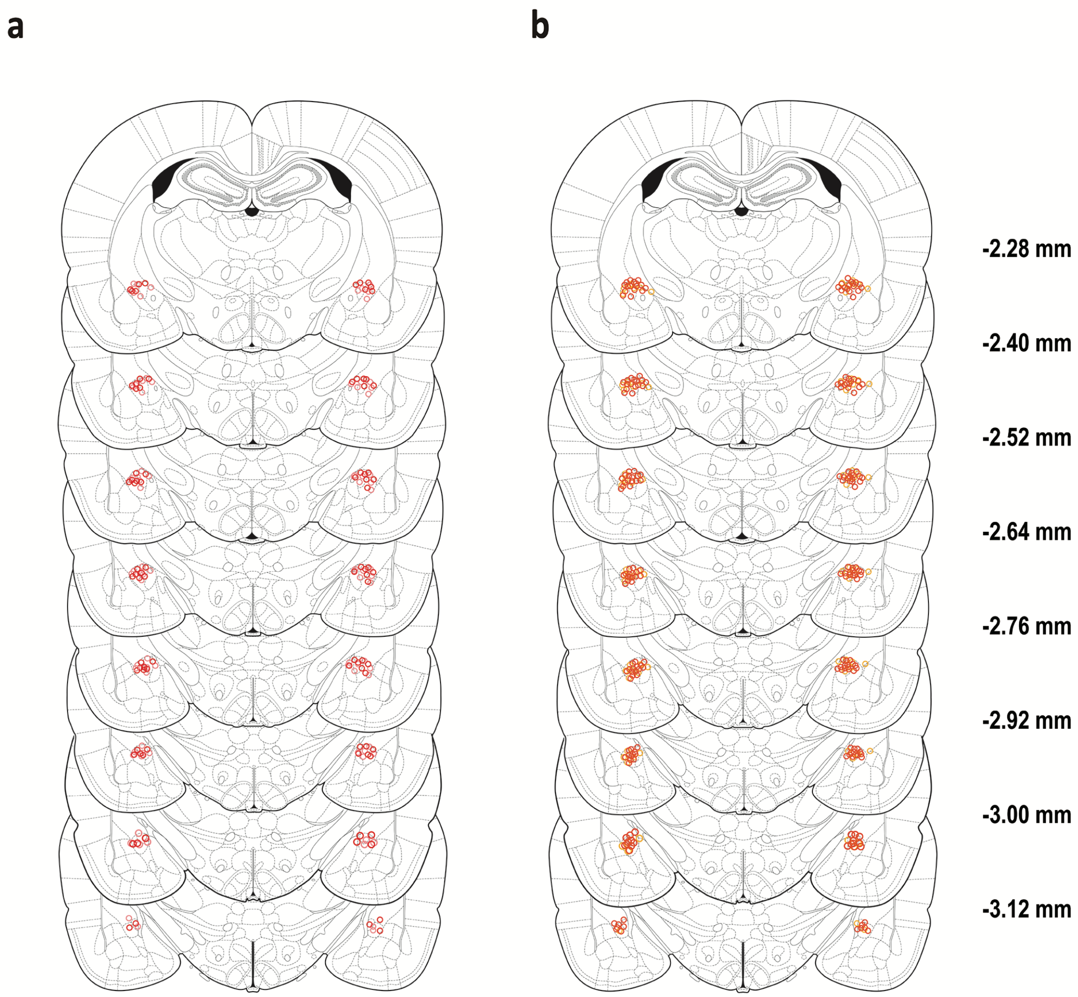


Propranolol group, CeA cannula placement (N=7)
Vehicle group, CeA cannula placement (N=7)

hM4Di group, CeA cannula placement (N=15)
mCherry group, CeA cannula placement (N=7)

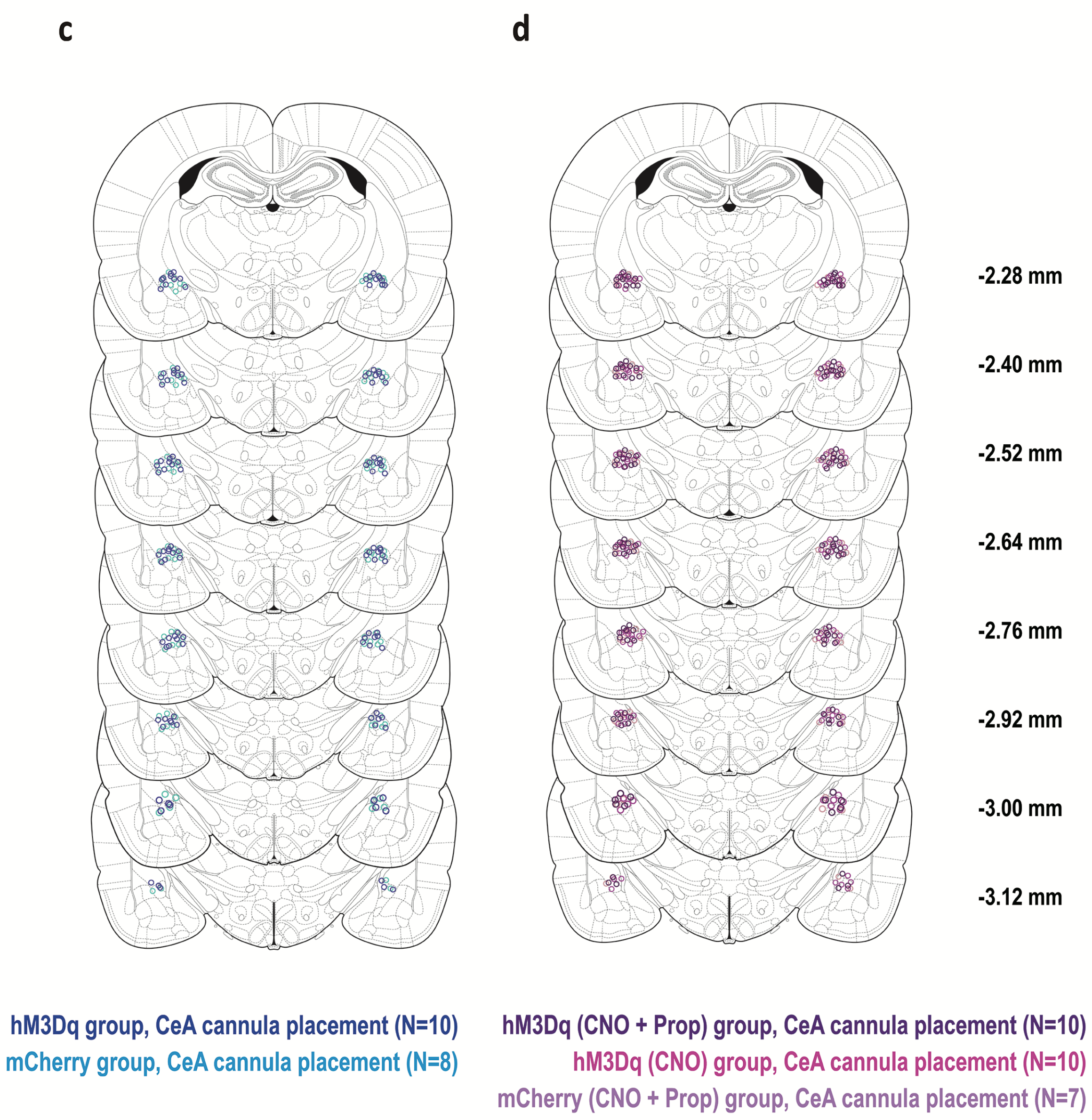


**Supplementary Figure 1. Virus and CeA cannulation targeting for all experiments. Related to Figure 4 and 5.**

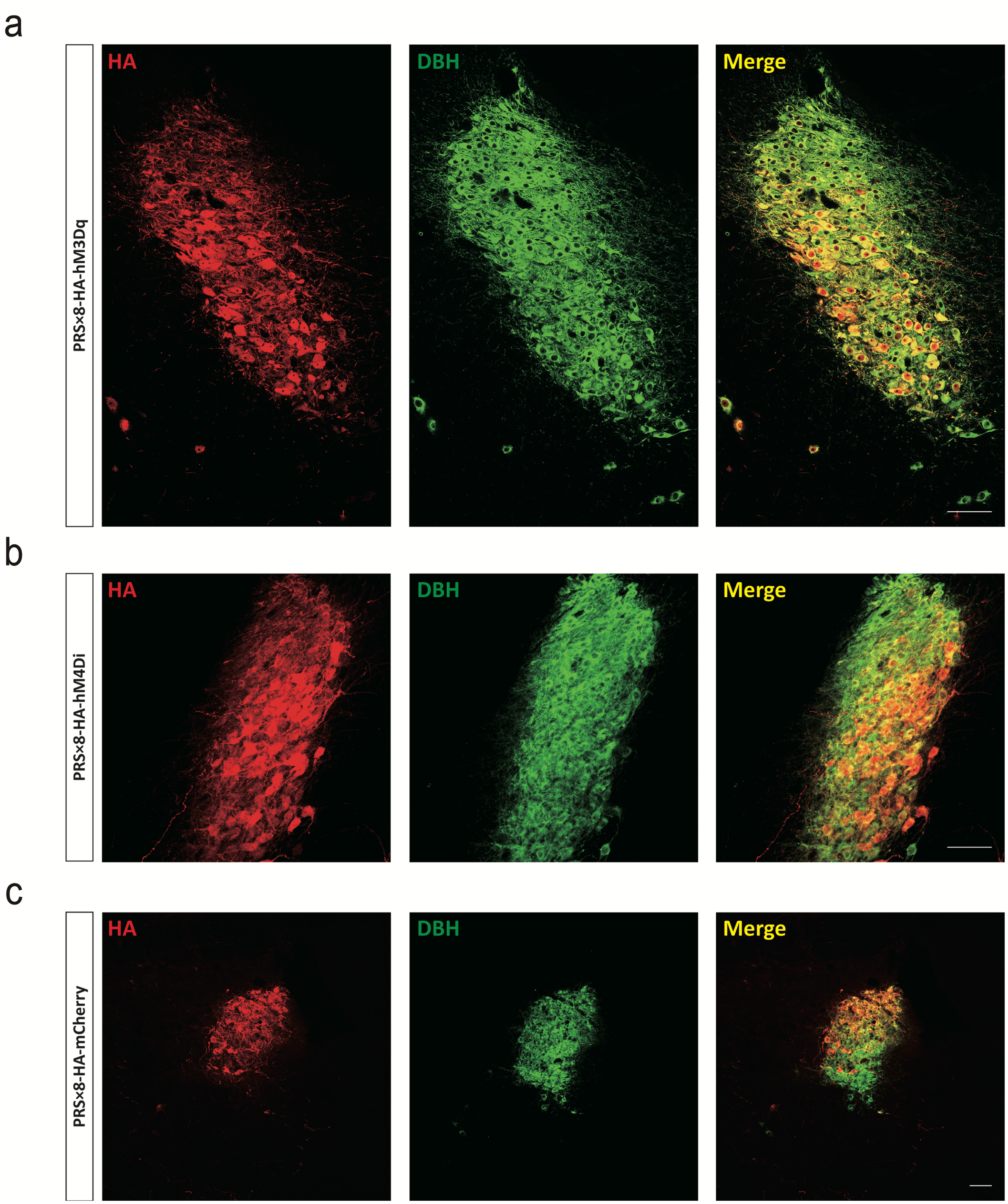


**Supplementary Figure 2. Representative image of DBH$^+$ and HA co-localization in hM3Dq/hM4Di LC rats. a.** Animals injected in LC with AAV-PRS×8-HA-hM3Dq and (**b**) PRS×8-HA-hM4Di show immunoreactivity for HA exclusively in DBH$^+$ neurons. **C.** Animals injected with PRS×8-HA-mCherry (control virus) in LC show mCherry expression only in DBH$^+$ neurons. Scale bars, LC panels = 50 μm, respectively.

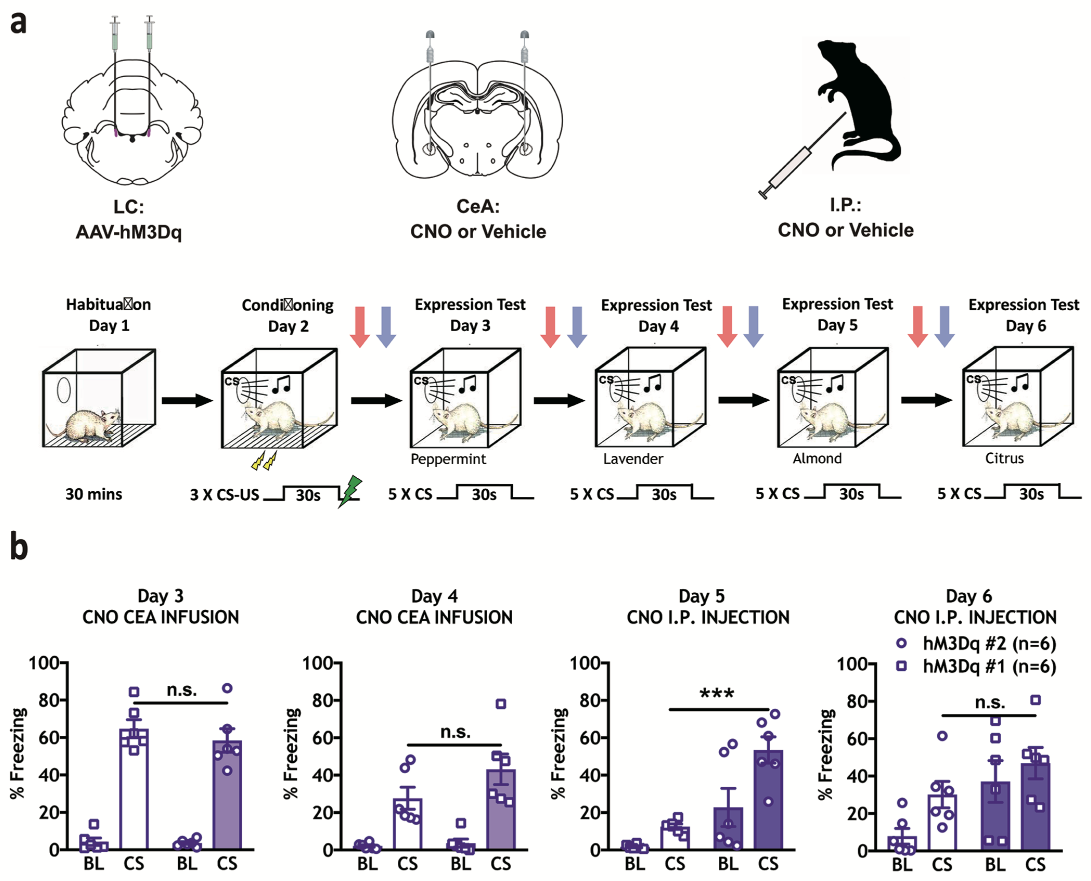


**Supplementary Figure 3. Experiments using axon terminal manipulations require longer incubation periods.**

**a.** Timeline indicating habituation (Day 1), mild conditioning (0.4 mA US) (Day 2), within subject expression test (CeA infusion) (Day 3, Day 4) and within subject expression test (i.p. injection) (Day 5, Day 6) phases. Three weeks following bilateral hM3Dq virus injection in LC, all rats (n=6/group) were trained using a moderate protocol and vertical arrow indicates either CNO (red) or vehicle (blue) treatment strategy. **b.** No significant difference between hM3Dq (CNO) v.s. hM3Dq (vehicle) within CeA infusion on Day 3 and Day 4. Within-subject analysis showed a significant difference in freezing between hM3Dq (CNO) vs. hM3Dq (vehicle) with systemic treatment (Day 5) ($t(10) = 5.694$, ***$p = 0.0002$). All error bars indicate mean ± SEM. ***$p < 0.001$

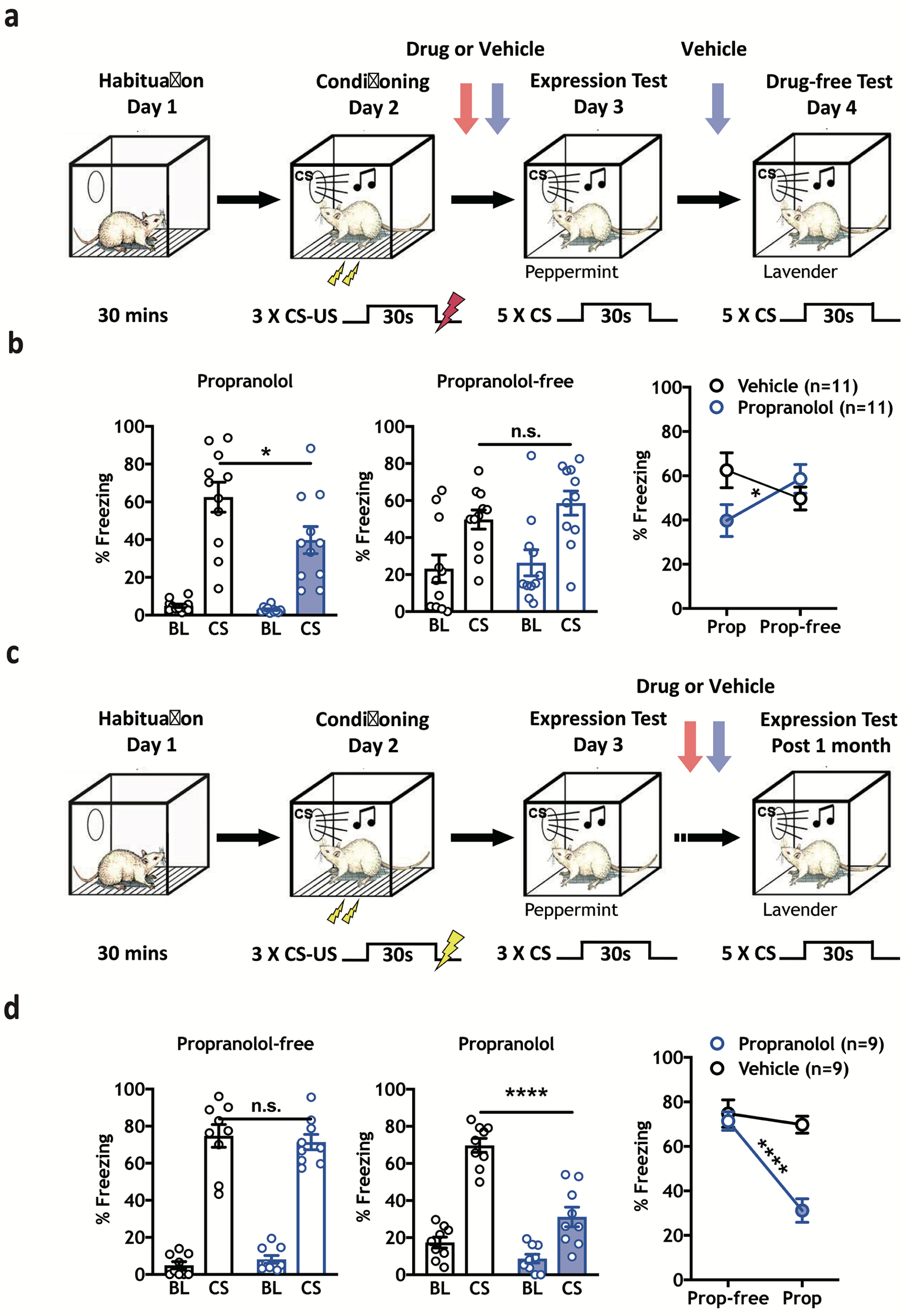


**Supplementary Figure 4. β-ARs are required for Pavlovian defensive responses, regardless of US intensity or memory age. a.** Experimental timeline for strong conditioning paradigm (1.0 mA US). **b.** Propranolol (10 mg/kg) reduced CS-elicited freezing levels during the expression tests compared to vehicle control animals (left panel: n = 11/group, *P = 0.0459), with no effect

observed between groups during a drug-free test (center panel, $p = 0.2973$, n.s.). A within-subject comparison of propranolol versus propranolol-free treatment on CS freezing followed by a post-hoc test revealed a significant difference in CS-elicited freezing for propranolol treatment day versus propranolol-free day (two-way RM ANOVA test, Interaction: $F(1, 20) = 9.669$, $^{**}p = 0.0055$; Drug: $F(1, 20) = 0.7270$, $p = 0.4039$; Time (Drug vs. Drug-free): $F(1,20) = 0.3610$, $p = 0.5547$; Sidak MCS, $^{*}p < 0.05$ for propranolol treated animals, n.s. for vehicle-treated animals between days). **c.** Timeline of remote Pavlovian threat conditioning (tested one month after conditioning (0.6 mA US)). **d.** Animals showed memory retention after one day, with no difference between groups (left panel, Day 3, n = 9/group; $p = 0.6548$). Subsequent treatment with propranolol after one month (10 mg/kg, Post one month) significantly attenuated CS-elicited freezing ($^{****}p < 0.0001$). Within-subject analysis showed a significant difference between a drug-free test (Day 3) freezing and freezing with propranolol treatment (Post one month); two-way RM ANOVA test, Interaction: $F(1, 16) = 14.06$, $^{**}p = 0.0017$; Drug: $F(1, 16) = 16.55$, $^{***}p = 0.0009$; Time (Drug-free vs. Drug): $F(1,16) = 23.21$, $^{***}p = 0.0002$; Sidak MCS, $^{****}p < 0.0001$ for propranolol treated animals, n.s. for vehicle-treated animals between days. All error bars indicate mean ± SEM. $^{*}p < 0.05$, $^{**}p < 0.01$, $^{***}p < 0.001$, $^{****}p < 0.0001$.

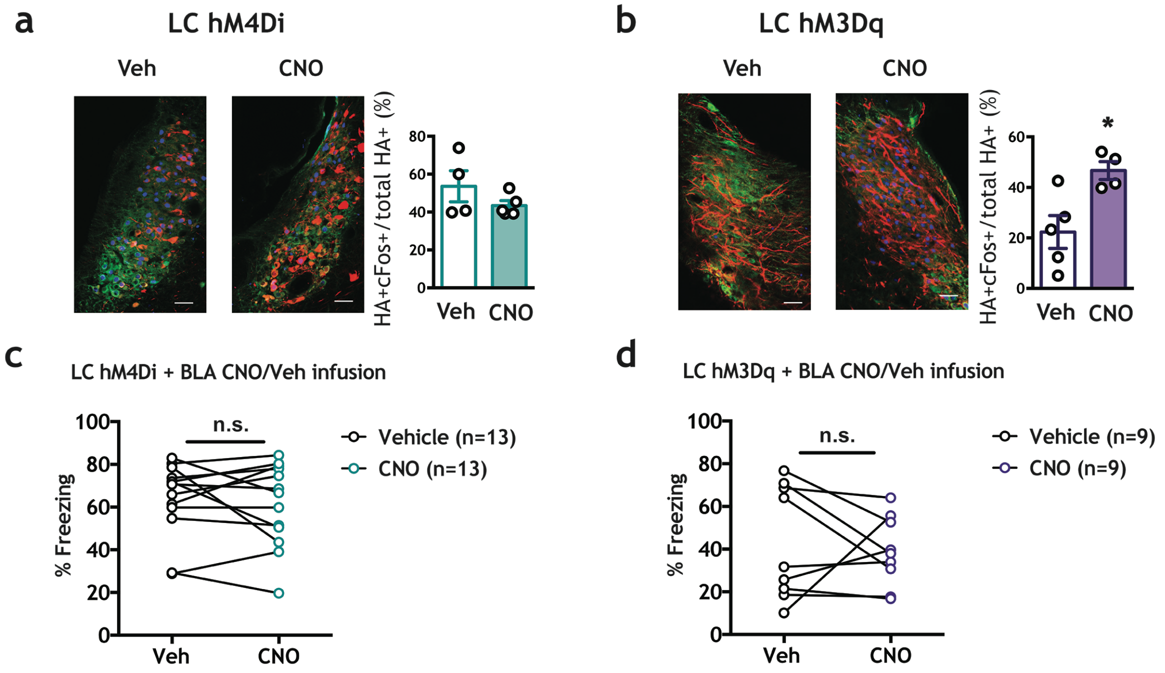


**Supplementary Figure 5. Control experiments to validate DREADD functionality and rule out noradrenergic BLA contributions to expression of conditioned freezing. a.** Representative images of LC in animals expressing hM4Di and receiving i.p. saline ("Veh", Left) or 5.0 mg/kg CNO (Middle) 30 minutes prior to footshock and 120 minutes prior to sacrifice (Antibody pseudocolors: Red=HA-tag, Green=DBH, Blue=c-Fos; Scale bars = 50 µm). Ratios of c-Fos+HA double labeled cells in LC over total HA cells in LC for saline and CNO conditions are shown (Right). **b.** Representative images of LC in animals expressing hM3Dq and receiving i.p. saline (Left) or 1.0 mg/kg CNO (Middle) 90 minutes prior to sacrifice. Ratios are shown for c-Fos+HA double labeled cells in LC over total HA cells in LC (Right, $*p = 0.018$). **c.** Expression of conditioned freezing during long-term memory testing for hM4Di-expressing animals receiving 0.3 µL/side saline or 1.0 mM/0.3 µl/side of CNO in basolateral amygdaloid complex (BLA). **d.** Expression of conditioned freezing for hM3Dq-expressing animals receiving 0.3 µL/side saline or 1.0 mM/0.3 µl/side of CNO in BLA.
